# Investigating the structure and bioactivity of lanthanum coordination compounds

**DOI:** 10.55730/1300-0527.3778

**Published:** 2025-12-24

**Authors:** Shantanu KADAM, Bhushan DHALE, Bapu YAMGAR, Swaminath BHATTAR

**Affiliations:** 1 Department of Chemistry, R.P. Gogate College of Arts and Science and R.V. Jogalekar College of Commerce (Autonomous), Ratnagiri, Maharashtra, India; 2 Department of Physics, R.P. Gogate College of Arts and Science and R.V. Jogalekar College of Commerce (Autonomous), Ratnagiri, Maharashtra, India; 3 Department of Chemistry, Dapoli Urban Bank Senior Science College, Dapoli, Maharashtra, India

**Keywords:** Lanthanum metal complexes, analytical techniques, elemental microanalysis, thermogravimetric analysis, antifungal studies

## Abstract

Lanthanum metal complexes were synthesized using a classical condensation method by reacting lanthanum nitrate salts with a bidentate ligand in a 1:2:1 molar ratio (M:L:L). The formation and structure of the resulting complexes were confirmed through a combination of analytical techniques, including Fourier transformation infrared spectroscopy, thermal gravimetric analysis, X-ray diffraction, elemental microanalysis, and scanning electron microscopy.

The lanthanum complexes synthesized exhibited strong in vitro antimicrobial activity. Antibacterial screening showed inhibition zones of 18–26 mm against *Staphylococcus aureus* and *Escherichia coli*, surpassing those of the reference drug ciprofloxacin (16–20 mm). Antifungal studies demonstrated inhibition zones of 15–23 mm against *Candida albicans* and *Aspergillus niger*. The MIC values ranged from 25 to 50 μg/mL, indicating high potency. Additionally, moderate antimalarial activity was observed against *Plasmodium falciparum* (IC_50_ = 12.4 μM).

These findings confirm that lanthanum complexes possess potent antibacterial and antifungal properties, highlighting their promise as potential candidates for novel antimicrobial and antimalarial drug development.

## 1 Introduction

The coordination chemistry sector focusing on lanthanum(III) ions, also known as rare earth metal ions, is quickly evolving as it finds applications in fundamental and applied research in diverse fields including chemistry, material science, and biology [1]. The electron configuration and contraction of lanthanum play significant roles in determining the structures of complexes, which can pose difficulties in managing the coordination environment of metal ions [2]. The lanthanums are a group of 15 rare earth elements that span from cerium to lutetium in the periodic table, with atomic numbers 58 to 71. Some experts also include lanthanum (atomic number 57) in this group, which comes before them in the periodic table, and the series is named after lanthanum. This series is characterized by a sequence of elements where the f orbital is either partially or completely filled with electrons, while the outer orbital remains vacant [3–6].

Within the lanthanum series, there exists a series of consecutive elements where the f orbital is either partially or filled with electrons, while the outer orbital remains unoccupied [5]. These elements exhibit oxidation states of +3 in their compounds, with some elements showing states of +2 and +4 as well. Notably, the +3 state is the most stable in lanthanum, gadolinium, and lutetium due to the vacancy of the 4f orbital, which undergoes half-filling before reaching complete filling [7]. Despite being classified as transition elements, these metals possess unique properties that distinguish them from other elements [6–10].

The coordination of organic chromophores with lanthanum (Ln) complexes gives rise to intriguing luminescent properties. This can be attributed to the excitation of the ligand moiety, which sensitizes the f–f transition luminescence. Notably, these complexes exhibit sharp bands that consistently appear at specific wavelengths, regardless of the surrounding atmosphere. Consequently, these complexes have garnered significant interest as versatile tools for luminescent devices, bioprobes, sensors [11–15], organic-light emitting diodes [16,17], time-resolved microscopy [18], and photovoltaic coating and anticancer agents [19–24].

Lanthanide complexes have several uses in medical investigation, diagnosis, and treatment, and they are unquestionably bioavailable. Their application in cancer treatment and therapy is growing because of their chemical and magnetic characteristics [25]. Lanthanide complexes with natural ligands exhibit enhanced antimicrobial and antioxidant activities compared to their free ligands or metal salts. Europium, dysprosium, and gadolinium complexes with caffeic and p-coumaric acids show stronger activity against *Escherichia coli*, *Bacillus subtilis*, and *Candida albicans*. Complexes of erbium, terbium, dysprosium, and holmium with 3-bromo-5-iodobenzoic acid display moderate activity against *C. albicans* and *Staphylococcus aureus*, and high activity against *E. coli*. Similarly, samarium and terbium complexes with aminobenzoic and 2-amino-5-chlorobenzoic acids show greater antimicrobial effects than the free ligands [26–29].

The uncontrolled growth of abnormal cells is an inherent characteristic of the cancer development process. This disease ranks as the second most common cause of death globally, resulting in millions of deaths each year [30]. Some Ln(III) complexes have been identified for their significant biological roles, such as binding to DNA and displaying antioxidant properties [19].

The efficiency of 1,10-phenanthroline as a sensitizer for lanthanum ions is attributed due to its capability to absorb and effectively transfer energy to the excited states of Ln^3+^ ions [31–33]. Quinoline and its derivatives, on the other hand, are recognized as a class of remarkably luminous compounds. These compounds possess the ability to coordinate through both oxygen and nitrogen atoms, allowing for a diverse coordination mode. Moreover, they have proven to be effective in bridging lanthanum ions. Notably, a significant amount of research has been conducted on 8-hydroxyquinoline and its derivatives, which have demonstrated intriguing properties when used as ligands for lanthanum metals [34–36]. Aromatic carboxylic acid ligands feature multiple coordination sites, facilitating the coordination of lanthanum ions with the oxygen atoms of these ligands in diverse coordination modes. As a result, lanthanum organic coordination complexes with varied structures can be synthesized. The aromatic carboxylic acid ligands are particularly beneficial as they can enhance the fluorescence of lanthanum ions by circumventing the forbidden 4f–4f transition caused by spin and parity restrictions, thereby leading to relatively weak luminescence intensity [37].

The main goal of the present research project was to prepare lanthanum complexes and investigate their critical structural characteristics and biological activities. The complexes were characterized using a variety of techniques including elemental analysis, Fourier transform infrared spectroscopy (FTIR), thermogravimetric differential thermal analysis (TG-DTA), scanning electron microscopy (SEM), X-ray diffraction (XRD), and fluorescence studies. The prepared complexes were then tested for their antibacterial activity against two gram-positive bacteria (*Streptococcus pyogenes* and *S. aureus*) and two gram-negative bacteria (*E. coli* and *Pseudomonas aeruginosa*). They were also tested for their antifungal activity against three types of fungus (*C. albicans*, *A. niger*, and *Aspergillus clavatus*) and for their antimalarial activity against *Plasmodium falciparum*.

The TG-DTA revealed stepwise thermal decomposition, indicating the formation of intermediate species and providing insights into the thermal stability and decomposition mechanism of the complexes. XRD confirmed their crystal structure and molecular identity, while fluorescence studies showed efficient intramolecular energy transfer from the ligand to the lanthanum(III) ion, explaining their luminescence. The complexes exhibited strong antibacterial, antifungal, and antimalarial activities, surpassing those of several standard drugs. Overall, the results highlight the potential of lanthanum(III) complexes as promising therapeutic agents, warranting further in vivo and mechanistic studies to assess their safety and efficacy.

## 2 Experimental

### 2.1. Materials and methods

All chemicals and solvents utilized were of analytical grade. Lanthanum nitrate hexahydrate, 1,10-phenanthrolin, and 8-hydroxy quinoline were purchased from Research Lab Fine Chem Industries (Mumbai, India), while nicotinic acid was obtained from Loba Chemie Pvt. Ltd. (Mumbai, India) and utilized as received. The infrared spectra were recorded in KBr pellets on a Shimadzu spectrometer. Elemental analyses were performed on a JEOL JSM-IT200 elemental analyzer. XRD analysis of the material was conducted using an Ultima IV X-ray diffractometer. The material’s fluorescence study was carried out using a Jasco spectrometer at room temperature. TG-DTA was executed using a METTLER TOLEDO instrument under air atmosphere.

Antibacterial strains, namely *E. coli* (MTCC 443), *P. aeruginosa* (MTCC 1688), *S. aureus* (MTCC 96), and *S. pyogenes* (MTCC 442), and antifungal strains, namely *C. albicans* (MTCC 227), *A. niger* (MTCC 282), and *A. clavatus* (MTCC 1323), were procured from the Institute of Microbial Technology, Chandigarh [MTCC stands for Microbial Type Culture Collection and Gene Bank].

### 2.2. Preparation of La(1,10-phen)_2_(8-Hq)

**Figure 1:**
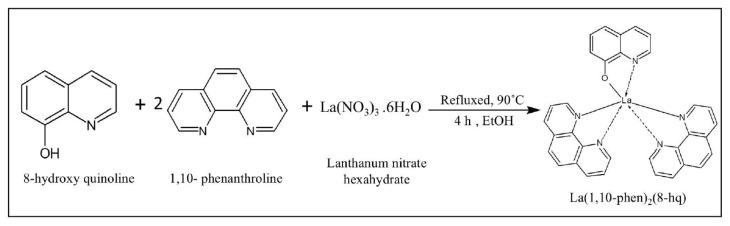
Preparation of the complex (8-hydroxy quinoline) bis (1,10-phenanthrolin) lanthanum La(1,10-phen)_2_(8- Hq).

The process for synthesizing La(1,10-phen)2(8-Hq) is illustrated in Figure 1. The compound was obtained by reacting the ligands 8-hydroxy quinoline (8-Hq), 1,10-phenanthrolin (1,10-phen), and lanthanum nitrate hexahydrate (metal ligand) in a molar ratio of 1:2:1 in ethanol. To prepare the solution, 0.15 g (0.001 mol) of 8-Hq and 0.40 g (0.002 mol) of 1,10-phen were dissolved in 60 mL of absolute ethanol in a 100-mL three-neck flask. The mixture was stirred in an inert atmosphere at 90 °C for 2 h. Subsequently, it was cooled to 70 °C, and a solution of lanthanum nitrate hexahydrate (0.43 g, 0.001 mol) in 3 mL of deionized water was added dropwise. After being stirred for an additional 2 h, a yellowish precipitate of the complex formed. The precipitate was separated from the reaction mixture, filtered, and then dried at 90 °C in an oven for 24 h.

## 3 Results and discussion

### 3.1. Structural characterization of material (8-hydroxy quinoline) bis (1,10-phenanthrolin) lanthanum La(1,10-phen)_2_(8-Hq)

**Figure 2:**
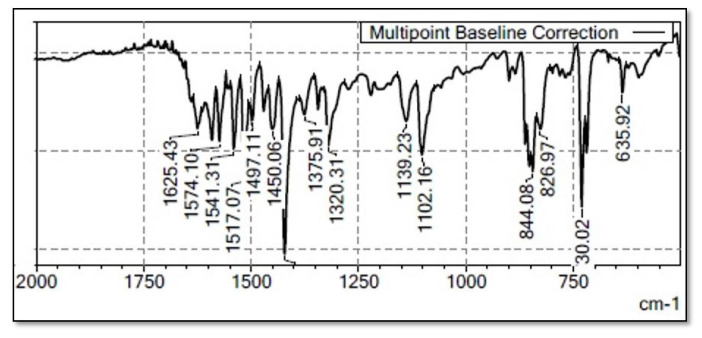
FTIR spectra of the complex (8-hydroxy quinoline) bis (1,10-phenanthrolin) lanthanum.

The material’s characterization was conducted using spectroscopic techniques, specifically FTIR. From Figure 2, the absorption observed in the mid-wavenumber region (1600–500 cm^–1^) corresponds to the in-plane and bending vibrations of the heavy atom (La). On the other hand, the absorption below 1000 cm^–1^ in the low wavenumber region is attributed to out-of-plane modes. The bands observed at 1600–1577 cm^–1^ are assigned to the stretching vibration of C=C from the quinoline ligand.

The C=C/C=N stretching associated with the pyridyl group in La(1,10-phen)_2_(8-Hq) can be observed at 1500. The sharp peak at 1321 cm^–1^ is a result of C–N vibrational absorption. In the case of La(1,10-phen)_2_(8-Hq) and La(1,10-phen)_2_(NA), the vibrations at 1375 cm^–1^ are attributed to the C=C/C=N stretching in the quinoline fragments. Additionally, the presence of quinolinic rings is indicated by the characteristic peaks ranging from 600 to 800 cm^–1^ [37–39].

### 3.2. Scanning electron microscopy and energy-dispersive X-ray analysis

**Figure 3:**
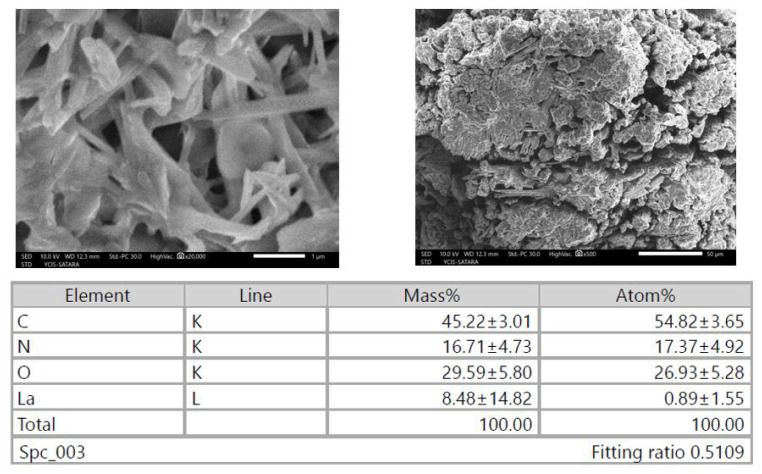
SEM and elemental images of the complex (8-hydroxy quinoline) bis (1,10-phenanthrolin) lanthanum.

The surface morphology of lanthanum complexes is examined by employing a focused electron beam to scan the surface. From Figure 3, the resulting SEM images exhibit a distinctive dumbbell and rod-like appearance with a smooth and polished aspect, showing the crystallinity of the complexes. To determine the chemical composition of the Ln(III) complexes, energy dispersive X-ray spectroscopy (EDX) is utilized. This analytical technique enables elemental analysis and chemical characterization of the samples. Additionally, the EDX analysis of the complexes provides valuable information regarding the presence of lanthanum, carbon, oxygen, and nitrogen elements. Notably, the EDX profile of the La(III) complexes displays peaks corresponding to C, O, and Ln(III) elements, thereby corroborating the proposed structure [40].

### 3.3. X-ray diffraction studies

**Figure 4:**
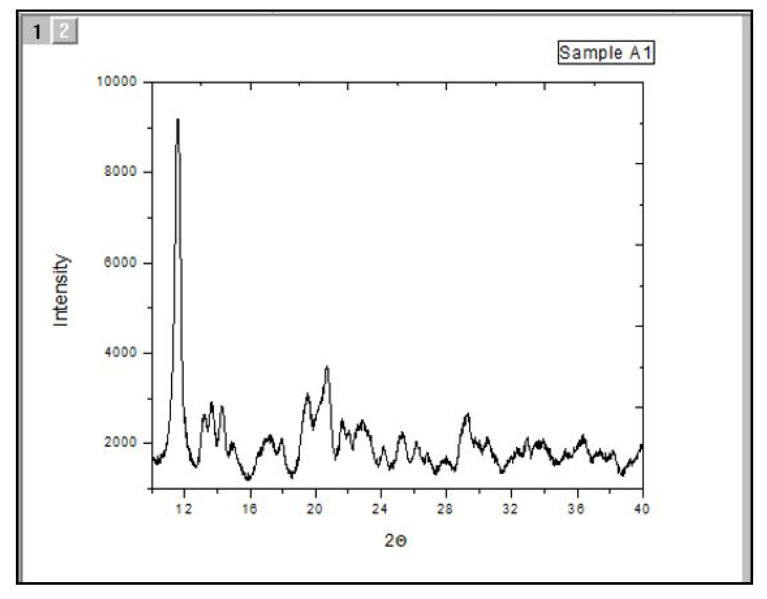
XRD pattern of the complex (8-hydroxy quinoline) bis (1,10-phenanthrolin) lanthanum.

The flexible nondestructive analytical method known as XRD is used to examine the phase composition, crystal structure, and orientation of powder, solid, and liquid samples, among other physical parameters. Determining the purity of synthesized chemicals is mostly dependent on their XRD examination. To guarantee precise outcomes, every XRD pattern was acquired after the compounds underwent an array of processes, including washing, filtering, air-drying, and being exposed to air for a minimum of 1 week. Figures 4 show the XRD patterns of the synthesized La(1,10-phen)_2_(8-Hq) compounds [40–42].

### 3.4. Fluorescence study

**Figure 5:**
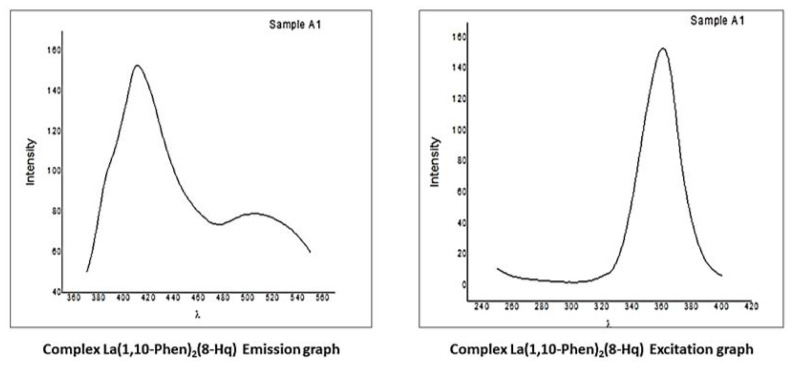
Luminescence spectra of the complex (8-hydroxy quinoline) bis (1,10-phenanthrolin) lanthanum.

The coordination of lanthanum complexes with organic chromophores results in a fascinating luminosity. This occurs because the excitation of the ligand moiety sensitizes the f–f transition luminescence, resulting in distinct bands that remain constant regardless of the surrounding atmosphere. As a result, these complexes have been extensively studied as they have excellent potential in luminous devices, bioprobes, and sensors. The emission spectrum of the complex La(1,10-phen)_2_(8-Hq) recorded at 410 nm (excitation spectrum 360 nm) is shown in Figure 5 [43–46].

### 3.5. Thermogravimetric differential thermal analysis

The TG-DTA was conducted under nitrogen atmosphere, with a heating rate of 10 °C/min, within the temperature range 33–1000 °C for both complexes. The thermal analysis of the lanthanum complexes exhibited a multistage disintegration or loss process, which occurred in two distinct steps, indicating a remarkable level of thermal stability. Initially, the loss of water molecules located outside the coordination sphere took place, followed by subsequent stages that led to a gradual decrease in weight and the release of specific compounds from the prepared complexes. The temperature ranges at which these stages occurred varied depending on the nature of the ions involved in complex formation. The remaining material after the completion of the dissociation process may correspond to the lanthanum oxide that constitutes the complex.

**Figure 6:**
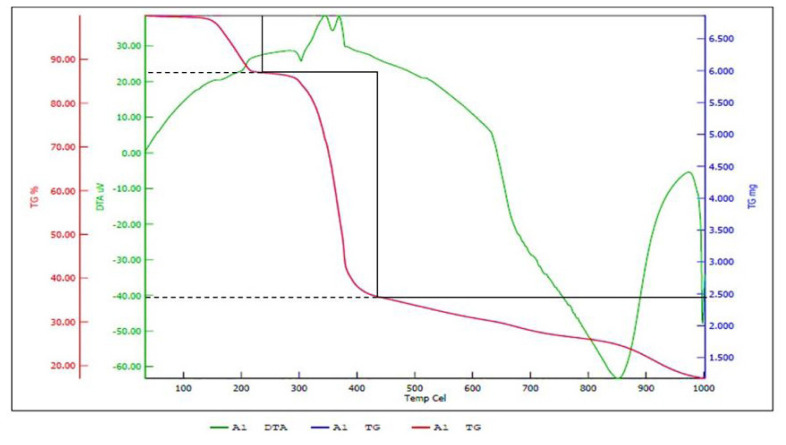
TGA thermograms of (8-hydroxy quinoline) bis (1,10-phenanthrolin) lanthanum.

In Figure 6, the temperature ranges from 240 to 430 °C, resulting in a gradual decomposition of the complex and a decrease in mass of approximately 52% due to the elimination of a coordinated molecule [47–49].

### 3.6. Biological studies

Studying the biological activity of lanthanum complexes is fundamental in the quest for safe and potent therapeutic drugs to treat various diseases such as bacterial infections and cancer. Lanthanum complexes are employed as models in biology to study the structure of biomolecules and biological processes [50–54]. The antifungal and antibacterial properties of the compounds investigated were analyzed by studying their microbial activities. Minimum inhibitory concentration (MIC) determination by broth dilution involves preparing dilutions of the test compound in broth medium and inoculating them with the target microorganism. After incubation, the lowest concentration inhibiting visible growth is noted as the MIC. The MIC in micrograms per milliliter (μg/mL) was used to express the investigated microorganism’s antibacterial and antifungal activities. Man’s personal comforts and conveniences rely largely on the control of the microbial population, which is essential to prevent the spread of illness, infection, decomposition, pollution, and spoilage caused by them. The antibacterial activity of the complexes was examined using two strains of gram-positive bacteria, namely *S. pyogenes* and *S. aureus*, as well as two strains of gram-negative bacteria, *E. coli* and *P. aeruginosa* [55,56]. According to Table 1, the study’s findings revealed that the substance had remarkable antibacterial properties against both gram-positive and gram-negative bacteria. Further study indicates that the complexes possess antibacterial properties, effectively inhibiting the growth of bacteria by blocking their active routes. The effectiveness of each compound against bacteria was determined by conducting tests with a variety of standards, such as ampicillin, ciprofloxacin, norfloxacin, chloramphenicol, and gentamycin (Table 1). Additionally, the antifungal potential of the compounds was evaluated using nystatin and griseofulvin as reference standards (Table 2). The current study demonstrated comparable findings regarding the antimicrobial efficacy of the complex [57–60].

All the prepared complexes were screened for antimalarial activity in the Microcare Laboratory & TRC (Surat, Gujarat, India). The in vitro antimalarial assay was conducted in 96-well microtiter plates using the microassay methodology by Rieckmann et al. *P. falciparum* 3D7 strain cultures were maintained in RPMI 1640 medium supplemented with HEPES, D-glucose, sodium bicarbonate, and heat-inactivated human serum. Parasites were synchronized to obtain ring stage cells. A ring stage parasitaemia of 0.8% to 1.5% at 3% haematocrit was prepared and maintained uniformly. Test samples were prepared in DMSO and diluted to obtain concentrations ranging from 0.4 μg/mL to 100 μg/mL. Chloroquine served as the reference drug. After incubation, smears were prepared and stained to observe parasite maturation. The concentration inhibiting complete maturation into schizonts was recorded as MIC. The antimalarial activity against standard drugs (chloroquine and quinine) and complexes is shown in Table 3 [61–67].

**Figure 7.1:**
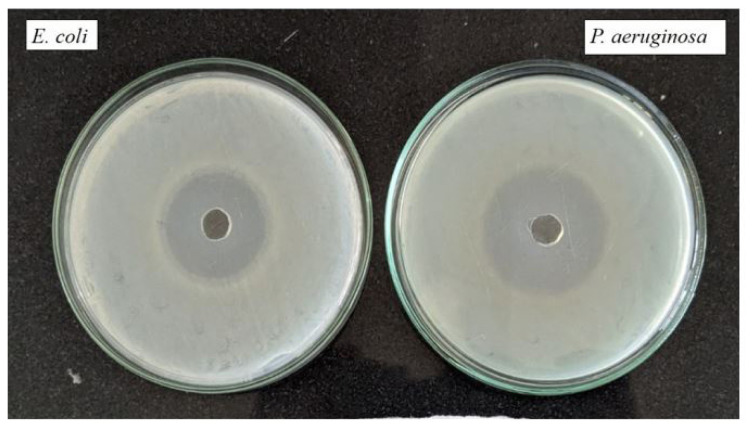
Gram-negative strain against (8-hydroxy quinoline) bis (1,10-phenanthrolin) lanthanum.

**Figure 7.2:**
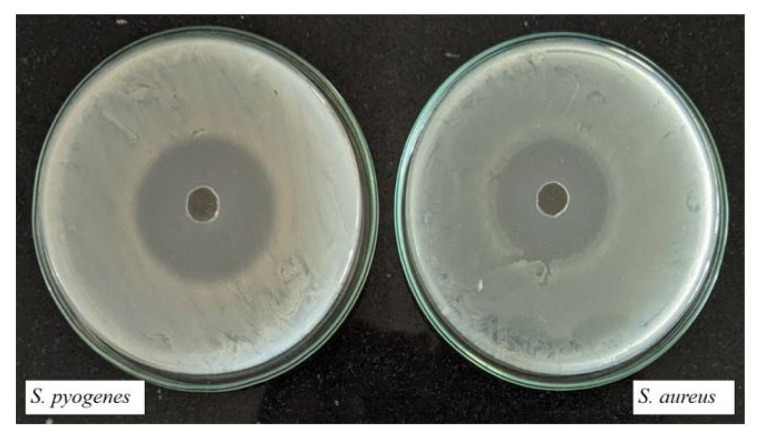
Gram-positive strain against (8-hydroxy quinoline) bis (1,10-phenanthrolin) lanthanum.

Figures 7.1 and 7.2 show the antibacterial activity evaluation using the agar well (or disk) diffusion method against different bacterial strains. The plates are inoculated with *E. coli*, *P. aeruginosa*, *S. pyogenes*, and *S. aureus*. The central well/disk contains the test sample. The clear circular region around the well, known as the zone of inhibition, indicates suppression of bacterial growth due to the antimicrobial action of the sample. The presence of distinct inhibition zones against both gram-negative bacteria (*E. coli* and *P. aeruginosa*) and gram-positive bacteria (*S. pyogenes* and *S. aureus*) demonstrates that the test sample exhibits broad-spectrum antibacterial activity.

## 4 Conclusion

Lanthanum complexes were synthesized from lanthanum nitrate hexahydrate, 1,10-phenanthroline, and 8-hydroxy quinolone and characterized using elemental analysis, FTIR, SEM-EDX, and TG-DTA.

The TG-DTA results revealed stepwise thermal decomposition, indicating the formation of various intermediate species. The data provided insights into the thermal stability and decomposition mechanism of the complexes. Stability and decomposition patterns were found to depend on the coordination environment of the lanthanum ion.

XRD confirmed the crystal structure and molecular identity of the complexes.

Fluorescence spectroscopy showed efficient intramolecular energy transfer from the ligand’s triplet state to the lanthanum(III) excited state, explaining the observed luminescence.

The complexes exhibited strong antibacterial properties, inhibiting microbial growth by disrupting cellular functional pathways. Antibacterial, antifungal, and antimalarial assays demonstrated that the complexes performed better than several standard drugs against the pathogens tested.

The findings indicate the potential of lanthanum(III) complexes as novel therapeutic agents with antimicrobial and antimalarial activity. Further studies, including in vivo antioxidant testing, mechanistic investigations, and animal model experiments, are recommended to evaluate their safety and efficacy.

## Figures and Tables

**Table 1:** Antibacterial activity against standard drug and complex.

The standard drugs					
Minimal inhibition concentration [μg/mL]					
Sr. No.	Drug	*E.coli*	*P. aeruginosa*	*S. aureus*	*S. pyogenes*
		*MTCC 443*	*MTCC 1688*	*MTCC 96*	*MTCC 442*
1	Gentamycin	0.05	1	0.25	0.5
2	Ampicillin	30	--	40	25
3	Chloramphenicol	50	50	50	50
4	Ciprofloxacin	25	25	50	50
5	Norfloxacin	10	10	10	10
6	La(1,10-phen)_2_(8-Hq)	100	250	100	125

**Table 2:** Antifungal activity against standard drug and complex.

The standard drugs				
Minimal fungicidal concentration [μg/mL]				
Sr. No.	Drug	*C. albicans*	*A. niger*	*A. clavatus*
		*MTCC 227*	*MTCC 282*	*MTCC 1323*
1	Nystatin	100	100	100
2	Griseofulvin	500	100	100
3	La(1,10-phen)_2_(8-Hq)	250	500	1000

**Table 3:** Antimalarial activity against standard drugs and complex.

The standard drugs		
Antimalarial activity table		
Minimal inhibition concentration [μg/mL]		
Sr. No.	Drug	MEAN IC_50_ values
1	Chloroquine	0.020 μg/mL
2	Quinine	0.268 μg/mL
3	La(1,10-phen)_2_(8-Hq)	0.78 μg/mL
